# Specific alterations in plasma proteins during depressed, manic, and euthymic states of bipolar disorder

**DOI:** 10.1590/1414-431X20154550

**Published:** 2015-09-08

**Authors:** Y.R. Song, B. Wu, Y.T. Yang, J. Chen, L.J. Zhang, Z.W. Zhang, H.Y. Shi, C.L. Huang, J.X. Pan, P. Xie

**Affiliations:** 1 Department of Neurology, The First Affiliated Hospital of Chongqing Medical University, Chongqing, China; 2 Chongqing Key Laboratory of Neurobiology, Chongqing, China; 3 Institute of Neuroscience and the Collaborative Innovation Center for Brain Science, Chongqing Medical University, Chongqing, China

**Keywords:** Bipolar disorder, Plasma, Proteomic, Carbonic anhydrase 1, Apolipoprotein

## Abstract

Bipolar disorder (BD) is a common psychiatric mood disorder affecting more than 1-2% of the general population of different European countries. Unfortunately, there is no objective laboratory-based test to aid BD diagnosis or monitor its progression, and little is known about the molecular basis of BD. Here, we performed a comparative proteomic study to identify differentially expressed plasma proteins in various BD mood states (depressed BD, manic BD, and euthymic BD) relative to healthy controls. A total of 10 euthymic BD, 20 depressed BD, 15 manic BD, and 20 demographically matched healthy control subjects were recruited. Seven high-abundance proteins were immunodepleted in plasma samples from the 4 experimental groups, which were then subjected to proteome-wide expression profiling by two-dimensional electrophoresis and matrix-assisted laser desorption/ionization-time-of-flight/time-of-flight tandem mass spectrometry. Proteomic results were validated by immunoblotting and bioinformatically analyzed using MetaCore. From a total of 32 proteins identified with 1.5-fold changes in expression compared with healthy controls, 16 proteins were perturbed in BD independent of mood state, while 16 proteins were specifically associated with particular BD mood states. Two mood-independent differential proteins, apolipoprotein (Apo) A1 and Apo L1, suggest that BD pathophysiology may be associated with early perturbations in lipid metabolism. Moreover, down-regulation of one mood-dependent protein, carbonic anhydrase 1 (CA-1), suggests it may be involved in the pathophysiology of depressive episodes in BD. Thus, BD pathophysiology may be associated with early perturbations in lipid metabolism that are independent of mood state, while CA-1 may be involved in the pathophysiology of depressive episodes.

## Introduction

Bipolar disorder (BD) is a chronic, severe, and highly debilitating mental illness characterized by depressive and manic episodes. BD affects more than 1-2% of the general population of different European countries and contributes to increased health care expenditure (1). Although several hypotheses have been proposed to explain BD pathoetiology, its underlying molecular basis remains poorly understood. Currently, clinical diagnosis of BD is based upon the criteria of the Diagnostic and Statistical Manual of Mental Disorders, fifth edition (DSM-5), or International Classification of Diseases, 10th revision (ICD-10), which are somewhat subjective because they rely on behavioral observations. As a result, approximately 40% of BD patients are initially misdiagnosed with major depressive disorder (MDD) (2). Moreover, clinical outcome in the majority of BD cases is often poor with high relapse rates, chronicity, lingering residual symptoms, and cognitive and functional impairment (1). Therefore, an improved understanding of the molecular basis of BD may lead to objective lab-based diagnostic tools, and would be helpful in improving diagnosis for this disorder.

It is thought that a non-hypothesis-based approach will be effective in identifying novel biomolecules involved in the pathophysiology of disease states. Compared with genomic analyses, proteomic studies focus on the protein as the “biological effector molecule”, thereby avoiding inconsistencies in translational regulation (3). The majority of proteomic research on BD focuses on post-mortem brain tissue, which provides some insight into BD-associated differential protein expression (4). However, technical difficulties associated with different post-mortem interval times and variable factors (e.g., pH) complicate these findings. Previous studies have shown that brain imbalances are reflected in the peripheral circulation, possibly because of regulatory interactions between the nervous and immune systems (5). Moreover, plasma is an easily accessible diagnostic sample with a minimal collection risk/cost profile. Thus, peripheral blood provides a more practical means by which to investigate BD pathophysiology.

Previously, Behan et al. (6) detected differentially expressed proteins in BD patients compared with healthy controls, although it remains unclear if these proteomic alterations are stable and exist independently of mood state. Several differentially expressed proteins are present in remission states, after the illness episode, and may reflect chronic changes (5), yet to our knowledge, there have been no previous studies comparing proteomic expression across varying mood states of BD. Therefore, we performed a differential proteomic study based on two-dimensional electrophoresis (2-DE) coupled to matrix-assisted laser desorption/ionization-time-of-flight/time-of-flight tandem mass spectrometry (MALDI-TOF/TOF MS), on plasma protein expression in BD subjects with depressed, manic, and euthymic mood states, in reference to healthy controls. We used immunoblotting to validate expression of the differentially expressed proteins, and performed biological network analysis on these proteins using Metacore (GeneGo, USA). Our findings may lead to improved understanding of the pathophysiological mechanisms underlying BD.

## Material and Methods

### Ethics statement

Written informed consent was obtained from all participants after a detailed description of the study was provided. The Ethics Committee (Institutional Review Board) of Chongqing Medical University approved this study, and the procedures employed for sample collection and analysis. All clinical investigations were conducted according to the principles expressed in the Helsinki Declaration.

### Subjects and samples

Bipolar subjects were enrolled from the Psychiatric Center of The First Affiliated Hospital at Chongqing Medical University (Chongqing, China). Diagnoses were performed using the Structured Clinical Interview from the DSM-IV-Axis I (SCID-I). Only BD type-I patients were enrolled. Manic and depressive symptoms were assessed using the Bech-Rafaelsen Mania Rating Scale (BRMS) and 17-item version of the Hamilton Depression Rating Scale (HDRS), respectively. Subjects were deemed euthymic if they scored <6 on both BRMS and HDRS scales and had been in remission with no significant symptoms or alterations in medication status for at least one month. Bipolar subjects were recruited if they exhibited BRMS ≥9 (manic) or HDRS ≥16 (depressed). Individuals with other pre-existing physical or mental disorders, medication use, illicit drug use, and/or abnormalities in clinical laboratory tests (blood and urine examination, and liver function tests) were excluded. Healthy controls matched by age, gender, and body mass index (BMI) were recruited from the Medical Examination Center of The First Affiliated Hospital at Chongqing Medical University. Candidates with a family history of BD or other psychiatric, neurological, or medical history were excluded from the healthy control group. In total, 10 euthymic BD, 20 depressed BD, 15 manic BD, and 20 demographically matched healthy control subjects were recruited. Demographic and clinical information are reported in Table 1.

**Table:**
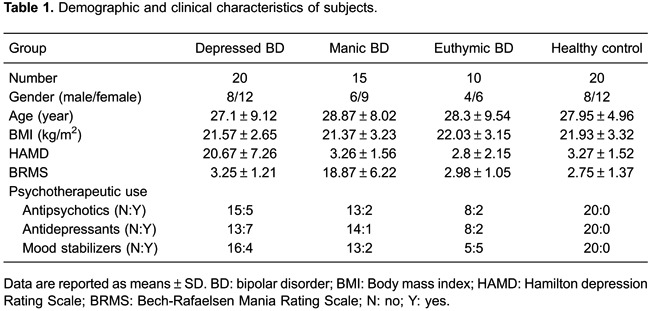

### Depletion of high-abundance plasma proteins

Fasting blood samples were collected before breakfast in 6 mL EDTA tubes (BD vacutainers catalog no. 367 863; Becton, Dickinson & Co., USA), and centrifuged at 1500 *g* for 15 min at room temperature within 1 h of collection. Plasma aliquots were then stored at -80°C until later analysis. Plasma samples were defrosted and equal volumes of plasma from the 4 groups (euthymic BD, depressed BD, manic BD, and healthy control) pooled. Following the manufacturer’s instructions, 420 µL volumes from each pool were depleted of the 7 most abundant plasma proteins (specifically, albumin, IgG, antitrypsin, IgA, transferrin, haptoglobin, and fibrinogen) using a multiple affinity removal system (MARS)-human 7 high-performance liquid chromatography (HPLC) column (4.6 mm inner diameter × 50 mm; Agilent, USA). Processed sample pools were purified by trichloroacetic acid (TCA) precipitation and then air-dried for 5 min. Proteins were dissolved in dissociation solution (7 mM urea, 2 M thiourea, 4% CHAPS, 50 mM DTT, 0.2% 3-10 Bio-Lyte; Bio-Rad Laboratories, USA), and measured using the Bradford method. Immediately before isoelectric focusing (IEF), the samples were further diluted to 100 μg/350 µL with dissociation solution.

### Two-dimensional electrophoresis (2-DE)

A total of 12 gels were developed, and each pooled sample was run in triplicate to control for gel variation. For the first-dimension IEF phase, 17 cm IPG strips [pH 3-10 nonlinear (NL), Bio-Rad] were used. After passive rehydration for a minimum of 12 h, the strips were focused and stained as previously described (7). Analytical gels were scanned using an Epson 10000XL scanner (Epson Co., Ltd., China) at an optical resolution of 600 dpi. Image analysis was performed according to a previously described procedure (7). Image analysis and spot detection were accomplished using Gaussian spot modeling with PD-Quest software version 8.0.1 (Bio-Rad Laboratories). Integrated intensities demonstrating at least 1.5-fold up- or down-changes were used to determine statistical differences in protein expression between each group (7).

### Protein identification by MALDI-TOF/TOF MS

Protein identification was performed according to a previously described procedure (7). MS integrated with MS/MS spectra were searched against the International Protein Index (IPI HUMAN V3.78, 86,392 entries) using GPS Explorer version 3.78 (Applied Biosystems, USA) and MASCOT version 2.1 (Matrix Science, USA). The search parameters were set according to the previously described procedure (7).

### Western blot analysis

Western blotting was performed using the same pooled samples in the initial 2-DE analysis. Individually, 15 depressed BD, 15 manic BD, 10 euthymic BD, and 15 healthy control samples were used. Equal amounts (5-15 µg) of plasma protein were separated on 6-12% SDS-PAGE gels and transferred to PVDF membranes. Membranes were blocked in 5% nonfat milk in Tris-buffered Saline (TBS) with 0.1% Tween-20 (TBS-T) for 1 h at room temperature, and then incubated overnight at 4°C with a primary antibody. After three TBS-T washes, membranes were then incubated with a peroxidase-conjugated secondary antibody for 1.5 h at room temperature. Membranes were washed three times with TBS-T, and the signal developed using Luminata™ Crescendo Western HRP Substrate (Millipore, USA). PVDF membranes were washed and stained with Coomassie Blue, and the 66 kDa band representing albumin used as the loading control. Each sample was analyzed in duplicate. The primary antibodies used were all obtained from Abcam (USA) and diluted as follows: anti-apolipoprotein A1 (Apo A1) goat polyclonal antibody (1:3000), anti-serum amyloid P (SAP) rabbit monoclonal antibody (1:3000), anti-kallistatin rabbit polyclonal antibody (1:3000), anti-complement factor H-related protein 1 (CFHL1) rabbit polyclonal antibody (1:3000), anti-carbonic anhydrase 1 (CA-1) rabbit monoclonal antibody (1:5000), anti-apolipoprotein L1 (Apo L1) rabbit polyclonal antibody (1:1500), and anti-alpha 2 macroglobulin (A2M) mouse monoclonal antibody (1:2000). The secondary antibodies used were diluted as follows: peroxidase-conjugated goat anti-mouse IgG (1:7500; Kirkegaard & Perry Laboratories, USA), peroxidase-conjugated goat anti-rabbit IgG (1:7500; Bioworld, USA), and peroxidase-conjugated rabbit anti-goat IgG antibody (1:7500; ZSGB-Bio, China). After washing, antibody-detected protein bands were visualized by enhanced chemiluminescence (ECL) and exposed to film. After immunodetection, membranes were stained with Coomassie Blue as an internal control. Western blot signals were densitometrically quantified using Quantity One software (Bio-Rad). The relative intensity of each protein was normalized to the total protein input in each lane and a healthy control to obtain fold-change values.

### Network and functional analysis by MetaCore

Gene symbols for the identified plasma proteins were uploaded into Metacore (version 6.6, GeneGo) for biological network construction. The “GeneGo Pathway Maps” algorithm in MetaCore was used to construct hypothetical networks of uploaded proteins. Relevant pathway maps were then ranked based on statistical significance with the uploaded data sets.

### Statistical analysis

Statistical analysis was performed using the Statistical Package of Social Science (SPSS, IBM, USA) for Windows (version 19.0). Data are reported as means±SD. Student’s *t*-tests were used to analyze significant differences between two groups within PD-Quest. All tests were two-tailed. Analysis of variance (ANOVA) was used to identify proteins with significant expression differences across the 4 groups in Western blotting. Our Western blot results followed a normal distribution. Statistical significance was set at P<0.05.

## Results

### Subjects

There were no significant differences in gender, age, or BMI in the depressed, manic, and euthymic BD groups compared to the healthy control group (Table 1).

### 2-DE and MALDI-TOF/TOF MS

**Figure 1:**
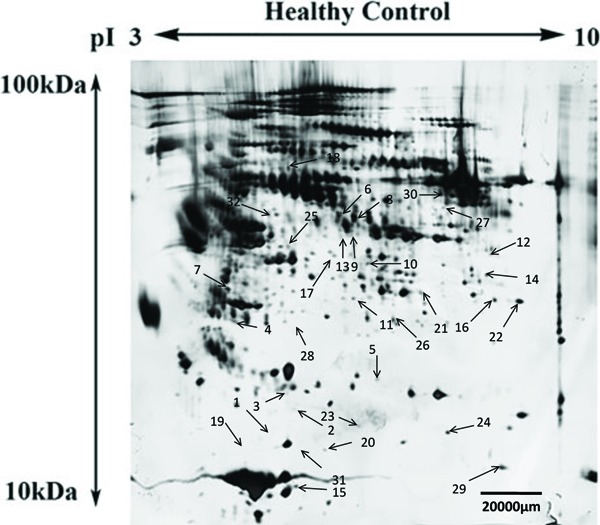
Representative image of a two-dimensional electrophoresis silver stained gel of depleted plasma sampled from healthy control subjects. Thirty-two differentially expressed protein spots (numbered with arrows) were identified across various bipolar disorder (BD) mood states (depressed BD, manic BD, and euthymic BD) relative to healthy controls.

For all samples, a mean of 875±22 spots was detected by PD-Quest, and 718±25 spots matched, with an average matching rate of 82.1%. Student’s *t*-tests showed that the intensities of 105 distinct protein spots changed more than 1.5-fold in one or more BD mood states compared with healthy controls. In the depressed BD group, 33 spots were up-regulated and 31 spots down-regulated. In the manic BD group, 20 spots were up-regulated and 17 spots down-regulated, while in the euthymic BD group, 49 spots were up-regulated and 31 spots down-regulated. Overall, the 105 differential protein spots corresponded to 32 mutually exclusive proteins, indicating that some spots correspond to protein isoforms (Figure 1). The proteins were all identified by MALDI-TOF/TOF MS (Table 2). Of the 32 mutually exclusive proteins, 16 were differentially expressed independent of mood state in the depressed, manic, and euthymic BD groups compared with the healthy control group. In addition, 16 proteins were changed in only one mood state (7 in the depressed BD group, 7 in the manic BD group, and 4 in the euthymic BD group) compared with the healthy control group (Supplementary Table S1).

**Table:**
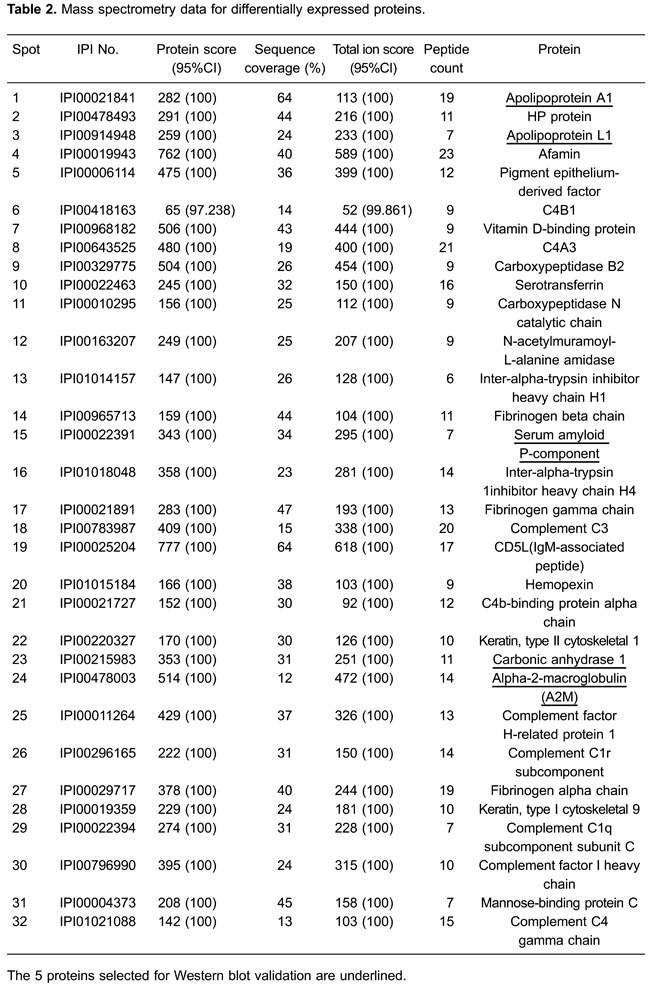

### Altered biological networks in BD plasma

To identify altered biological networks associated with deregulated plasma proteins in BD, we used MetaCore analysis to illustrate putative biological linkages between the 32 mutually exclusive proteins. The top-ten related canonical pathway maps and GeneGo cellular and molecular processes networks are listed (Table 3). Notably, the most significant biological network identified was the immunoresponsive lectin-induced complement pathway (P=1.065×10^−27^). Similarly, the inflammatory complement system was revealed to be the most significant GeneGo cellular and molecular processes network (P=2.802×10^−35^).

**Table:**
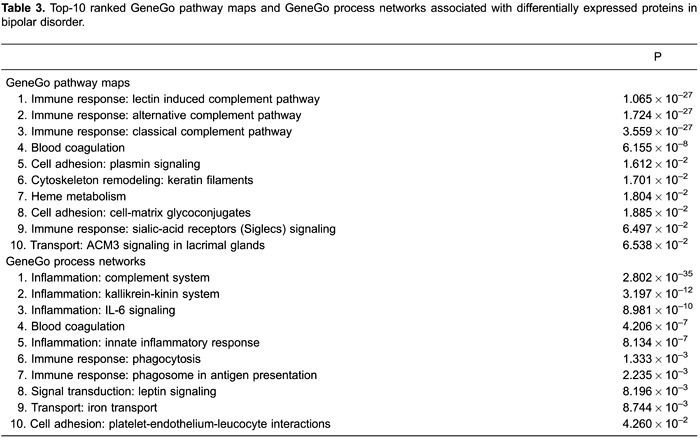

### Western blot validation

**Figure 2:**
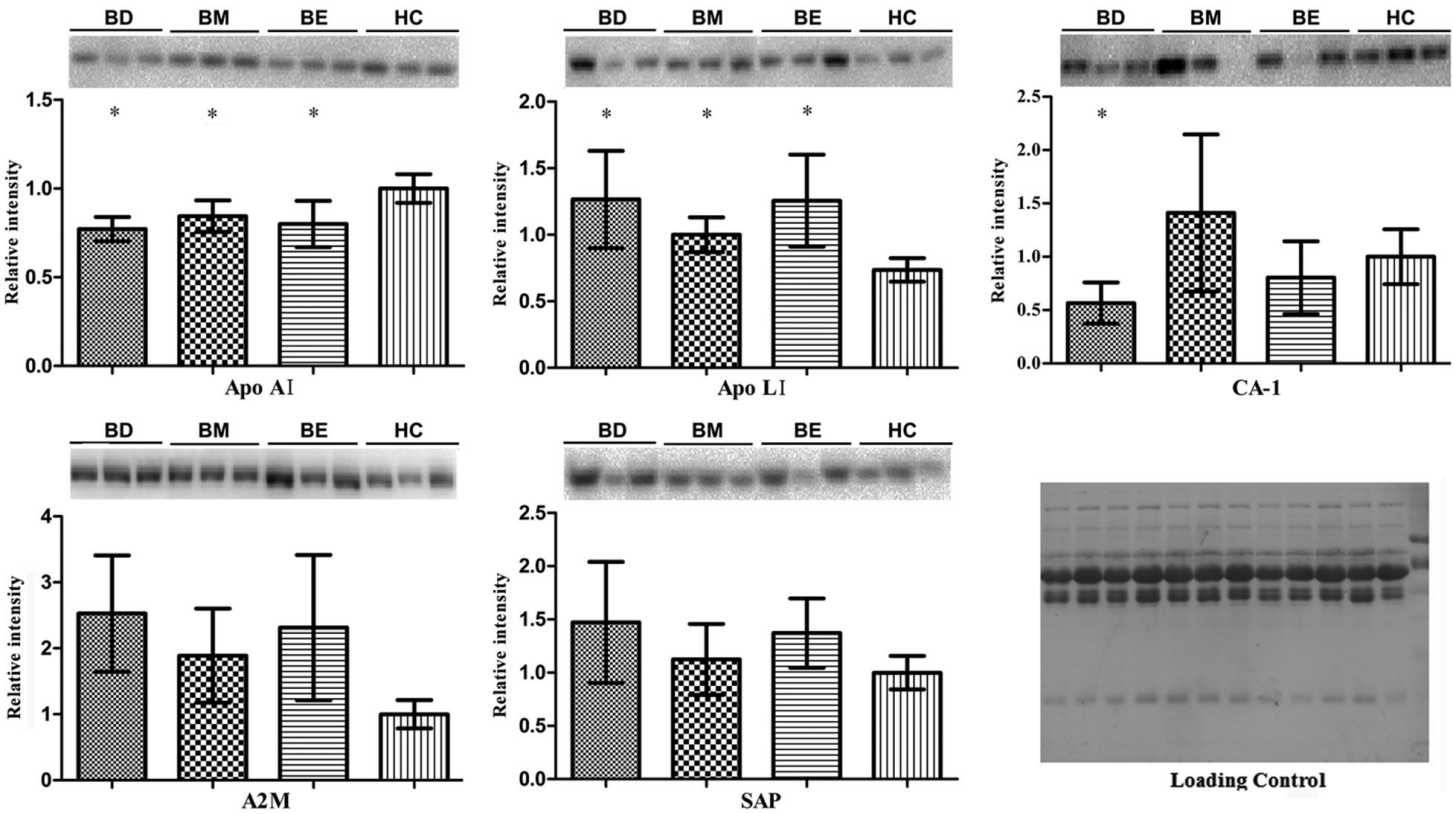
Western blotting of Apo A1, Apo L1, CA-1, A2M, and SAP. Proteins were analyzed in individual samples from healthy control subjects (HC, n=15) and bipolar disorder patients: BD: bipolar depression (n=15); BM: bipolar mania (n=15); BE: bipolar euthymia (n=10). All samples were analyzed in duplicate. Quantitative analysis of protein bands was performed using the Quantity One software (Bio-Rad, version 4.6.7). Data are reported as means±SD. A representative subset of blots is presented for each protein. The Western blot results followed a normal distribution and were analyzed by one-way analysis of variance (ANOVA). Relative intensity of each protein was normalized to the total protein input of each lane (loading control), and fold-change was obtained and compared to a healthy control. *P<0.05, compared to HC (ANOVA).

Based on the close relationship between inflammation and neurogenesis in the disease process, we selected 5 proteins (SAP, Apo A1, CA-1, Apo L1, and A2M) for Western blot validation using crude non-depleted plasma samples. Western blot assays were initially performed with the same pooled plasma samples used for 2-DE analysis. Coomassie staining of membranes after Western blotting showed similar intensities for the 66-kDa albumin band in samples from all 4 groups, indicating that similar protein amounts were loaded. All 5 proteins were detected by immunoblotting and displayed significant differences (P<0.05), thereby validating our MS/MS protein identification results. The 5 proteins were further validated in individual plasma samples from the 4 groups (Figure 2). Of these 5 proteins, Western blotting of Apo A1, Apo L1, and CA-1 confirmed the protein expression trends of our 2-DE analysis, and were statistically significant (P<0.05). However, differential expression of SAP was not statistically significant (P>0.05). This may be explained by either differences in the dynamic range between 2-DE and Western blotting, intrinsic variability associated with the procedural steps of proteomic and Western blotting analysis (8), major protein depletion for 2D-E, or individual variability. We attempted to examine A2M by Western blotting, but were not successful because of poor antibody performance.

## Discussion

Despite significant improvements in genetic, neurochemical, neuroimaging, and neuroanatomical techniques, little is known about the underlying molecular basis of BD. Its diagnosis remains symptom-based with a high misdiagnosis rate (9). These factors indicate the need to improve our understanding of the molecular alterations associated with BD. In this study, we used a differential proteomic approach based on 2-DE coupled with MALDI-TOF/TOF MS to compare the plasma proteomes in depressed, manic, and euthymic BD subjects relative to healthy controls.

To overcome variance in individual subjects, we pooled individual plasma samples collected from each group. Plasma is a difficult proteome to characterize because of its wide dynamic protein concentration range that exceeds the analytical capabilities of standard proteomic methods, and makes low-abundance protein detection challenging. Thus, removal of high-abundant proteins is crucial (10). Here, based on its strong reproducibility and specificity, we used the Agilent MARS to deplete high-abundance plasma proteins (11). Consequently, 2-DE resolution was improved, and a significant number of low-abundant protein spots visualized. Nevertheless, we were unable to replicate some of our proteomic findings in individual samples analyzed by immunoblotting. A similar discrepancy has been observed in other proteomic studies (8), and may be due to differences in the dynamic range between 2-DE and immunoblotting or intrinsic variability associated with the proteomic and immunoblotting procedures. Moreover, individual variability cannot be excluded, as BD is a complex mental disorder with a poorly understood pathoetiology.

In our differential proteomic experiments, we identified 105 protein spots, 32 of which were mutually exclusive (Table 2). Expression of 16 proteins was altered in BD compared with healthy controls, independent of mood state. Moreover, another 16 proteins were specifically altered in only one mood state compared with healthy controls, and thus, are specific to those mood states and may be associated with the molecular basis of specific BD mood states (Supplementary Table S1). To identify the biological networks associated with deregulated plasma proteins in BD, we used MetaCore (Table 3). Immunoregulation displayed the highest statistical significance. Increasing evidence suggests that chronic, mild inflammatory processes in the peripheral and central nervous system (neuro-inflammation) are involved in BD pathophysiology. BD is accompanied by: i) moderately increased plasma levels of pro-inflammatory cytokines, such as interleukin (IL)-6 and tumor necrosis factor-α; ii) increased protein and mRNA levels of IL-1β, and IL-1RA in the frontal cortex of post-mortem bipolar patients (12,13); and iii) increased acute phase protein levels, including haptoglobin and C-reactive protein (CRP), as well as complement factors, such as plasma C3 or C4 concentrations (12). Recently, Goldstein et al. (14) reviewed the literature and found 27 articles suggesting that inflammation and BD are linked through shared genetic polymorphisms and gene expression. It has also been hypothesized that certain inflammatory mediators are related to neuroprogression in BD (15,16). This corroborates the previous hypothesis that inflammatory mediators may be related to episode-related cognitive decline in BD patients (16). Furthermore, inflammation affects other neurotransmitter systems that may play important roles in inducing BD symptoms (17).

Of the 16 mood-independent differentially expressed proteins, Apo A1, Apo L1, and CA-1 achieved statistical significance in individual samples. Extensive evidence shows that cholesterol and apolipoprotein levels are disturbed in several psychiatric disorders, suggesting that the cholesterol system may lead to promising psychiatric biomarkers (18). Association between cholesterol and mental health has been tentatively explained on the basis of hypothesized neural mechanisms linking serum cholesterol to brain function. Cholesterol forms an integral part of cell membranes and is a major component of myelin. Furthermore, cholesterol plays a vital role in development, function, and stability of synapses (19). Beasley et al. (20) found significantly lower cholesterol levels in individuals with MDD or BD compared with controls. These investigators concluded that lower brain cholesterol levels lead to reduced synaptic density or function, and may be a pathophysiological feature of mood disorders. Chen et al. (21) found that after controlling for possible confounding factors, there were significant differences between high- and low-level high-density lipoprotein cholesterol (HDL-C) groups in depression scores, as well as various other symptoms of psychological distress. Subjects with lower serum HDL-C levels scored higher for depression, phobic anxiety, and somatization. Additionally, there is a strong positive relationship between cholesterol levels and cognition in schizophrenia (22). Overall, cholesterol disturbances in psychiatric disorders may strongly influence a patient’s mood state via synaptic stability and reduced serotonergic function.

The relationship between suicide and serum cholesterol has been the subject of much debate. Lester (23) published a meta-analysis exploring the link between low serum cholesterol levels and increased suicide risk. Follow-up studies found that individuals with lower cholesterol levels have a small, but statistically significant, increased risk of completing suicide (24,25). Moreover, individuals who have attempted suicide in the past have lower cholesterol levels, especially if violent methods were used. The relationship between suicide and cholesterol levels is further confirmed by the findings that the brains of violent suicide completers have lower gray-matter cholesterol content (25). There is also evidence that the relationship with serum cholesterol levels is much stronger for individuals with suicidal behavior characterized by violence and impulsivity (24). In our previous study, plasma levels of the apolipoproteins, low-density lipoprotein (LDL) and very-low-density lipoprotein (VLDL), were decreased in suicide attempters compared with non-attempters. Additionally, plasma cholesterol levels were decreased in suicide attempters relative to both non-attempters and healthy controls. Decreased plasma levels of unsaturated lipids have been observed in suicide attempters relative to both non-attempters and healthy controls (26). Findings from a double-blind pilot trial of 12 subjects (27), demonstrated a modest increase in impulsivity after a short course of cholesterol-lowering therapy that dissipated with a longer course of therapy. These findings reinforce the observations from cross-sectional studies.

Here, Apo A1 was down-regulated and Apo L1 up-regulated in BD subjects relative to healthy controls. Apo A1 is a constituent of the HDL fraction, and regulates plasma levels of free fatty acids, and has an important role in HDL and triglyceride-rich lipoprotein metabolism, and in the reverse cholesterol transport pathway (28). Apolipoproteins are altered in BD, schizophrenia, and other psychiatric disorders. Previously, we found that LDL and VLDL are the most prominent factors differentiating depressed patients from healthy controls, and that plasma unsaturated lipid levels are elevated in the depressed group (26). Interestingly, Apo A1 is unchanged in MDD, and may be a potentially useful biomarker for differential diagnosis of BD and MDD, although studies directly comparing MDD and BD patients will be required to test this (29). In agreement with our current study, Sussulini et al. found decreased Apo A1 levels in BD medicated patients, with levels restored to that of healthy controls in patients treated with lithium (3,28). Decreased peripheral Apo A1 levels were previously reported in schizophrenia patients, further corroborating our present results (30). Primarily associated with Apo A1-containing lipoproteins, Apo L1 is present on a subset of HDL particles, and positively correlates with plasma triglycerides (31). Additionally, previous studies have reported up-regulation of Apo L1 mRNA levels in schizophrenic post-mortem brain (32). Despite this evidence, the mechanisms underlying the association between apolipoproteins and psychiatric disorders remain unclear. However, with respect to our observed Apo A1 and Apo L1 changes, we can surmise that these lipid metabolism abnormalities are independent of mood state.

In contrast to the identified mood state-independent altered proteins, expression of CA-1, a carbonic anhydrase (CA) isoenzyme that catalyzes the CO_2_/HCO_3_ interconversion, was perturbed solely in the depressed BD group (33). Therefore, CA-1 down-regulation may be related to pathoetiology of depressed mood states in BD. Interestingly, Yolken’s group at Johns Hopkins demonstrated significantly increased CA-1 levels in brains from depressed individuals (34). Another CA isoform, CA-2, is expressed in glial cells, myelin, and the choroid plexus, and is a key driver of neuronal pH fluxes. Notably, CA inhibitors (e.g., acetazolamide, zonisamide, and topiramate) ameliorate depressive symptoms during the depressive phase of BD (35). To demonstrate that CA-1 is involved in the pathophysiology of depressive episodes in BD, it is necessary to show that CA-1 responds positively to symptomatic improvement. However, hospital referral rates of patients with depressive episodes who have received appropriate medication are very low, and we were unable to collect enough plasma samples from responders. Our future work will focus on the effect of antidepressants on plasma CA-1 levels.

The brain has the second highest concentration of lipids, exceeded only by adipose tissue. Both central and peripheral abnormalities in lipid metabolism and membrane dynamics have already been linked with a number of neurodegenerative and neuropsychiatric disorders, implying that lipid metabolism is of particular importance to brain function and dysfunction (36). Therefore, we have reason to assume that the peripheral changes in lipid metabolism identified in our study are significantly related to brain alterations in the patients. As it is difficult to obtain brain tissue samples in our clinical work, it is hard to intuitively discuss the relationship between peripheral and brain alterations. Subsequently, samples such as cerebrospinal fluid (CSF) provide an alternative way of examining the relationship between them. Accordingly, our group has collected CSF samples from patients with psychiatric disorders including BD, MDD, and schizophrenia. Proteomic and lipidomic research on these CSF samples will soon be performed, and we believe that further interesting discoveries will be made in the future.

We were unable to collect enough samples from drug-naive BD subjects to improve homogeneity. Most patients are under medication when admitted to hospital. Antipsychotics, antidepressants, and mood stabilizers are selected to treat BD, and different medications may induce different protein expression changes in the peripheral circulation. For example, expression of transthyretin tetramer significantly increased after two months of anti-psychotic treatment (37). Moreover, de Witte et al. (38) found that the anti-inflammatory cytokine, IL-10, responds to treatment in parallel with symptomatic improvement, and may be used as a potential treatment response biomarker in schizophrenia. Kati et al. (39) showed significantly higher serum malondialdehyde levels in patients with selective serotonin reuptake inhibitor intoxication compared with controls. Sussulini et al. (28) proposed Apo A-I as a candidate marker for response to lithium treatment at the serum protein level. Therefore, drug-naive subjects are usually enrolled when researchers aim to discover proteomic disease-related biomarkers, as the influence of drugs can be excluded. Correspondingly, most clinically used drugs reverse changes in target disease proteins. The strategy of enrolling medicated subjects may facilitate discovery of novel differential disease proteins beyond the targets of current drugs. Further work should focus on biomarker discovery using drug-naive BD subjects, followed by biomarker validation in enlarged study populations that includes medicated subjects. Finally, we were unable to collect enough information on the number of patients and controls using statins, yet statin use may be associated with lower depression risk through several possible underlying mechanisms (40). Further work will focus on the anti-depressant effects of statins in depressed BD (or some other aspect).

In this study using 2-DE coupled to MALDI-TOF/TOF MS, several differentially expressed proteins were identified in plasma sampled from BD subjects relative to healthy controls. A total of 16 proteins were altered in BD, independent of mood state, while 16 proteins were specifically associated with particular BD mood states. Two mood-independent differential proteins, Apo A1 and Apo L1, implicate early perturbations in lipid metabolism in BD pathophysiology. Moreover, down-regulation of a mood-dependent protein, CA-1, suggests it may be involved in the pathophysiology of depressive episodes in BD, although we were unable to determine if this change reflects mood state or medication. To develop BD biomarkers, further investigations on medication effects and other confounding factors are needed.

## Supplementary Material


