# Supplementary material for: Specific alterations in plasma proteins during depressed, manic, and euthymic states of bipolar disorder
Source: Braz J Med Biol Res. 2015 Sep 8;48(11):973–82. doi: 10.1590/1414-431X20154550 (PMC4671523; doi:10.1590/1414-431X20154550)
Supplement: Supplementary file 1 [file 1414-431X-bjmbr-48-11-00973-supp4550.pdf]

Table S1. Differentially expressed proteins in bipolar disorders (BD) identified by two-dimensional electrophoresis. The 5 proteins selected for Western blot validation are underlined**.** Spots were considered to be altered in specific groups if proteins were up-regulated or down-regulated 1.5-fold (less than 0.67 compared to controls) in only one BD state. Spots were altered across all BD groups if proteins were altered 1.5-fold across all three BD states. PI: isoelectric point; MW: molecular weight.

Spot	IPI No.	PI	MW (Da)	Depressed BD expression ratio (compared to control)	Manic BD expression ratio (compared to control)	Euthymic BD expression ratio (compared to control)	Healthy control expression ratio	Protein
**Altered across all BD groups**								
1	IPI00021841	5.56	30758.9	0.37	0.42	0.61	1	<u>Apolipoprotein A1</u>
2	IPI00478493	6.13	38940.5	4.29	2.56	2.48	1	HP protein
3	IPI00914948	5.58	42189.0	2.12	2.43	1.95	1	<u>Apolipoprotein L1</u>
4	IPI00019943	5.64	70962.7	2.11	1.82	2.20	1	Afamin
5	IPI00006114	5.97	46454.4	3.77	2.53	2.50	1	Pigment epithelium-derived factor
6	IPI00418163	6.89	194170.1	4.12	2.70	3.19	1	C4B1
7	IPI00968182	5.36	39969.3	2.49	3.51	0.04	1	Vitamin D-binding protein
8	IPI00643525	6.65	194218.1	4.01	1.91	3.76	1	C4A3
9	IPI00329775	7.61	48963.7	2.12	1.77	2.27	1	Carboxypeptidase B2
10	IPI00022463	6.81	79294.5	2.68	4.22	3.19	1	Serotransferrin
11	IPI00010295	6.86	52538.4	0.39	0.57	0.61	1	Carboxypeptidase N catalytic chain
12	IPI00163207	7.25	62748.1	0.25	0.35	0.44	1	N-acetylmuramoyl-L-alanine amidase
13	IPI01014157	6.31	101781.8	0.28	0.55	0.40	1	Inter-alpha-trypsin inhibitor heavy chain H1
14	IPI00965713	8.22	50436.4	1.54	3.46	3.12	1	Fibrinogen beta chain
15	IPI00022391	6.10	25485.2	1.80	0.04	0.65	1	<u>Serum amyloid P-component</u>
16	IPI01018048	5.72	76971.4	0.62	0.53	0.57	1	inter-alpha-trypsin 1inhibitor heavy chain H4
**Altered only in depressed BD group**								
17	IPI00021891	5.37	52106.1	2.21	1.42	1.16	1	Fibrinogen gamma chain
18	IPI00783987	6.02	188569.5	2.17	1.28	0.83	1	Complement C3
19	IPI00025204	5.28	39602.5	0.54	0.93	1.16	1	CD5L(IgM-associated peptide)
20	IPI01015184	6.45	29068.1	1.76	1.37	0.92	1	Hemopexin
21	IPI00021727	7.15	69042.2	0.63	0.83	1.05	1	C4b-binding protein alpha chain
22	IPI00220327	8.15	66170.1	1.57	1.21	1.00	1	Keratin, type II cytoskeletal 1
23	IPI00215983	7.14	28870.2	0.51	0.69	0.79	1	<u>Carbonic anhydrase 1</u>
**Altered only in manic BD group**								
24	IPI00478003	6.03	164613.4	1.16	0.19	0.80	1	<u>Alpha-2-macroglobulin (A2M)</u>
25	IPI00011264	7.38	38766.4	1.16	0.19	0.80	1	Complement factor H-related protein 1
26	IPI00296165	5.82	81606.4	0.81	0.31	1.25	1	Complement C1r subcomponent
27	IPI00029717	8.23	70227.0	0.72	0.55	0.71	1	Fibrinogen alpha chain
28	IPI00019359	5.14	62254.9	1.10	1.64	1.34	1	Keratin, type I cytoskeletal 9
29	IPI00022394	8.61	25985.2	0.76	1.66	0.88	1	Complement C1q subcomponent subunit C
**Altered only in euthymic BD group**								
30	IPI00796990	7.86	68976.0	1.03	1.01	1.71	1	Complement factor I heavy chain
31	IPI00004373	5.39	26526.2	1.20	0.81	2.79	1	Mannose-binding protein C
32	IPI01021088	6.70	189185.5	1.47	0.86	1.63	1	Complement C4 gamma chain
